# Cardiovascular and metabolic syndrome risk among men with and without erectile dysfunction: case-control study

**DOI:** 10.1590/S1516-31802010000300006

**Published:** 2010-05-06

**Authors:** João Paulo Zambon, Rafaela Rosalba de Mendonça, Marcelo Langer Wroclawski, Amir Karam, Raul D. Santos, José Antonio Maluf de Carvalho, Eric Roger Wroclawski

**Affiliations:** I MD. Urologist in the Health Review Program, Hospital Israelita Albert Einstein, São Paulo; attending urologist in the Micturition Dysfunction Group, Faculdade de Medicina do ABC (FMABC), Santo André, São Paulo, Brazil.; II MD. Urology resident at Faculdade de Medicina do ABC (FMABC), Santo André, São Paulo, Brazil.; III MD. Physician in the Renal Transplantation Group, Hospital Israelita Albert Einstein, São Paulo, Brazil.; IV MD. Urologist in the Health Review Program, Hospital Israelita Albert Einstein, São Paulo; Brazilian Institute of Cancer Control, São Paulo, Brazil.; V MD. Coordinator of the Lipid Clinic, Instituto do Coração (INCOR), São Paulo, Brazil; VI MD. Coordinator of the Preventive Medicine Center, Hospital Israelita Albert Einstein, São Paulo, Brazil.; VII MD, PhD. Chairman of Urology, Faculdade de Medicina do ABC (FMABC), Santo André; Head of the Department of Urology, Brazilian Institute of Cancer Control; and Urologist at Hospital Israelita Albert Einstein, São Paulo, Brazil. (In memoriam)

**Keywords:** Erectile dysfunction, Metabolic syndrome X, C-reactive protein, Cardiovascular diseases, Endothelium, Risk factors, Disfunção erétil, Síndrome X metabólica, Proteína C-reativa, Doenças cardiovasculares, Endotélio, Fatores de risco

## Abstract

**CONTEXT AND OBJECTIVE:**

Erectile dysfunction has been associated with cardiovascular diseases. The aim here was to evaluate cardiovascular risk through the Framingham Risk Score (FRS) criteria, C-reactive protein (CRP) assays and presence of metabolic syndrome (MS) in men with and without erectile dysfunction diagnosed within a healthcare program.

**DESIGN AND SETTING:**

A retrospective case-control study was conducted. The patients were selected from a healthcare program at the Hospital Israelita Albert Einstein, between January and December 2007.

**METHODS:**

222 men were retrospectively selected, and they were divided into two groups: men with erectile dysfunction (n = 111) and men without erectile dysfunction (n = 111). The patients were stratified according to the International Index of Erectile Function-Erectile Function domain (IIEF-EF domain). CRP and FRS were analyzed and the two groups were compared.

**RESULTS:**

The CRP levels were significantly higher among men with erectile dysfunction (P = 0.04). Patients with erectile dysfunction also had high FRS (P = 0.0015). CRP and FRS did not correlate with the severity of erectile dysfunction. The presence of metabolic syndrome was greater among men with erectile dysfunction (P < 0.05). The severity of erectile dysfunction was directly associated with metabolic syndrome.

**CONCLUSION:**

Men with erectile dysfunction presented higher cardiovascular risk according to the FRS criteria and CRP measurements. Severe erectile dysfunction seemed to have a correlation with metabolic syndrome.

## RESUMO

**CONTEXTO E Objetivo:**

Disfunção erétil está associada a doenças cardiovasculares. O objetivo foi avaliar o risco cardiovascular através dos critérios de Framingham (FRS), da dosagem de proteína C-reativa e da presença de síndrome metabólica em homens com e sem disfunção erétil diagnosticados em um programa de saúde.

**TIPO DE ESTUDO E LOCAL:**

Estudo retrospectivo tipo caso-controle foi realizado. Os pacientes foram selecionados de um programa de saúde do Hospital Israelita Albert Einstein, no período de janeiro a dezembro de 2007.

**MÉTODOS:**

222 homens foram retrospectivamente selecionados e divididos em dois grupos: homens com disfunção erétil (n = 111) e homens sem disfunção erétil (n = 111). Os pacientes foram estratificados de acordo com o Índice Internacional de Disfunção Erétil (International Index of Erectile Function, Erectile Function domain, IIEF-EF domain). A proteína C-reativa e o escore de Framingham foram analisados e os dois grupos foram comparados.

**RESULTADOS:**

O nível da proteína C foi significativamente maior entre homens com disfunção erétil (P = 0,04). Pacientes com disfunção erétil também tinham um escore de risco de Framingham alto (P = 0,0015). A proteína C e o escore de Framingham não se correlacionaram com o grau de gravidade da disfunção erétil. A presença de síndrome metabólica foi maior nos pacientes com disfunção erétil (P < 0,05). A gravidade da disfunção erétil esteve diretamente associada com a síndrome metabólica.

**CONCLUSÃO:**

Homens com disfunção erétil apresentaram maior risco cardiovascular de acordo com os critérios de Framingham e pela mensuração da proteína C-reativa. A disfunção erétil grave em homens parece ter correlação com a síndrome metabólica.

## INTRODUCTION

Erectile dysfunction is defined as the inability to achieve or maintain penile erection with sufficient quality to perform satisfactory sexual intercourse. It is not a direct threat to life, but should not be considered a benign disorder, because it has increasingly been associated with cardiovascular diseases such as ischemic cerebrovascular events, angina pectoris, myocardial acute failure and sudden death. Some authors have suggested that erectile dysfunction is a sentinel event and early marker of cardiovascular diseases.^1^

According to the Massachusetts Male Aging Study (MMAS), 52% of men aged between 40 and 70 years old have some degree of erectile dysfunction.^2^ Endothelial dysfunction and microvascular damage are involved in the pathogenesis of erectile dysfunction. Risk factors such as hypertension, smoking, obesity, diabetes mellitus and sedentary lifestyle are common in patients with coronary diseases and erectile dysfunction.^1,3^

The Framingham Risk Score (FRS) is widely recommended for assessing overall cardiovascular risk and estimating the 10-year risk of cardiovascular events. FRS evaluates clinical and laboratory findings such as history of smoking, obesity, diabetes mellitus, triglycerides, high-density lipoprotein (HDL) and fasting blood glucose levels.^4^

Ultrasensitive C-reactive protein (CRP) is a systemic inflammatory and cardiovascular biomarker that is associated with endothelial dysfunction and metabolic syndrome.^5^

Metabolic syndrome affects 10-25% of the adult population worldwide and is associated with atherosclerotic disease. The definition of metabolic syndrome includes the presence of at least three of the following items: male waist circumference greater than 102 cm, triglycerides greater than 150 mg/dl, HDL lower than 40 mg/dl, blood pressure greater than 130 × 85 mmHg and fasting glucose greater than 110 mg/dl.^6,7^ Reduction of smoking, regular exercise practices, healthy diet, weight reduction, diabetes control and changes in lifestyle are associated with lower incidence of erectile dysfunction and lower risk of metabolic syndrome.^8^

Some studies have supported the existence of an association between cardiovascular diseases and metabolic syndrome. The latter is a risk factor for endothelial dysfunction and vascular damage and there seems to be a relationship between erectile dysfunction and metabolic syndrome.^3,9,10^

## OBJECTIVE

The aim of this study was to evaluate cardiovascular risk through the FRS, ultrasensitive CRP measurement and the presence of metabolic syndrome, among men with and without erectile dysfunction who were diagnosed within a continuing health review program.

## MATERIAL AND METHODS

A total of 222 patients were retrospectively selected between January and December 2007 with ages ranging from 39 to 73 years. This was a case-control study. All of them were enrolled in the health review program of the Preventive Medicine Center of Hospital Israelita Albert Einstein. The patients were evaluated by a multidisciplinary team composed of general clinicians, cardiologists, dermatologists, urologists, ophthalmologists, physiotherapists, nutritionists, nurses and psychologists. These patients usually had yearly follow-up.

The men were divided into two groups according to presence or absence of erectile dysfunction and they were matched one-to-one according to their age. This study was approved by the Hospital Ethics Committee and an informed consent form was signed by all patients. Erectile function was evaluated in accordance with the International Index of Erectile Function — Erectile Function domain (IIEF-EF domain) score system. Men with IIEF-EF domain score lower than 10 were considered to present severe erectile dysfunction; if they had an IIEF-EF score between 11 and 16, they were classified as having moderate erectile dysfunction; an IIEF-EF score between 17 and 25 characterized patients with mild erectile dysfunction; an IIEF-EF score between 26 and 30 corresponded to no erectile dysfunction.^11^

Group 1 was composed of men without erectile dysfunction and group 2 by men with erectile dysfunction. The waist circumference, waist-hip index, triglycerides, high-density lipoprotein cholesterol (HDL), low-density lipoprotein cholesterol (LDL), total cholesterol, fasting blood glucose and blood pressure were measured. The presence of metabolic syndrome was determined in accordance with the National Cholesterol Education Program Adult Treatment Panel III^9^ criteria, and the two groups were compared.

The FRS, ultrasensitive CRP and metabolic syndrome were evaluated in both groups. Men with erectile dysfunction were divided again into three subgroups according to the degree of erectile dysfunction and the same parameters were evaluated in each subgroup.

The data were analyzed using the Statistical Package for the Social Sciences, version 11.0 (SPSS, Chicago, Illinois, United States) software program. The chi-square test was used to evaluate the associations between risk factors and erectile dysfunction. Multiple logistic regression analysis was performed to investigate the associations of variables with erectile dysfunction. Statistical significance was defined as p< 0.05.

## RESULTS

A total of 222 patients were enrolled in this study; 111 (50%) with erectile dysfunction and 111 (50%) without it. The mean age was similar in the two groups.

Hypertension, diabetes mellitus, smoking, obesity, body mass index, waist-hip ratio, resting heart rate, mean systolic blood pressure, mean diastolic blood pressure, total and LDL cholesterol, triglycerides and fasting blood glucose were higher in group 2 (men with erectile dysfunction). The clinical characteristics and laboratory findings can be seen in Table 1.

**Table 1.** Clinical characteristics and laboratory findings (mean and standard deviation) among patients according to the presence or absence of erectile dysfunction

	Group 1 Control	Group 2 Erectile dysfunction	P-value
Mean age (years)	51.04 ± 6.54	51.21 ± 6.86	0.886
Hypertension	23 (20.5%)	36 (32.7%)	< 0.001
Systolic blood pressure (mmHg)	127.18 ± 13.81	129.68 ± 15.98	0.380
Diastolic blood pressure (mmHg)	81.65 ± 7.2	84.5 ± 10.96	0.073
Resting heart rate (bpm)	69 ± 7	74.13 ± 8.02	< 0.001
High density lipoprotein cholesterol (mg/dl)	50.36 ± 12.56	46.20 ± 10.31	0.008
Low density lipoprotein cholesterol (mg/dl)	123.75 ± 35.85	122.39 ± 33.36	0.949
Cholesterol (mg/dl)	205.71 ± 40.52	202.32 ± 35.54	0.570
Triglycerides (mg/dl)	172.74 ± 122.56	185.75 ± 114.29	0.135
Diabetes mellitus	8 (7%)	28 (26.4%)	< 0.001
Fasting blood glucose (mg/dl)	94.42 ± 19.65	99.68 ± 39.91	0.807
Body mass index (kg/m^2^)	27.49 ± 3.6	28.27 ± 3.1	0.019
Waist-hip ratio	0.96 ± 0.59	1.0091 ± 0.95	< 0.001
Smoking	14 (12.5%)	30 (27.3%)	0.006

Men with erectile dysfunction presented higher cardiovascular risk, according to the CRP and FRS findings. The presence of metabolic syndrome was higher in this group. Cardiovascular risk and metabolic syndrome data can be seen in Table 2.

**Table 2.** Evaluation of cardiovascular risk and metabolic syndrome and erectile dysfunction

	Group 1: control	Group 2: men with erectile dysfunction	P
Framingham risk score	7.76 ± 3.31	11.41 ± 8.33	0.0015
C-reactive protein	1.7 (1.9-3.7)	2.1 (3.1-5.1)	0.04
Metabolic syndrome	29/82 (26.1%)	53/58 (52.3%)	0.002

The severity of erectile dysfunction was directly associated with metabolic syndrome, i.e. patients with severe erectile dysfunction presented a higher risk of having metabolic syndrome. This relationship is shown in Table 3.

**Table 3.** Metabolic syndrome and severity of erectile dysfunction

Metabolic syndrome	Severe erectile dysfunction	Moderate erectile dysfunction	Mild erectile dysfunction	P
Yes	10	14	29	0.005
No	5	22	31	

## DISCUSSION

According to the Massachusetts Male Aging Study (MMAS), 52% of men aged between 40 and 70 years old have some degree of erectile dysfunction.^2^ There is a relationship between systolic blood pressure, diabetes, obesity, smoking, dyslipidemia and erectile dysfunction, such that the prevalence of these risk factors is greater in patients with erectile dysfunction. Smoking appears to amplify the association between erectile function and other risk factors.^3^

The FRS is widely recommended for assessing overall cardiovascular risk, but there are large numbers of patients who have coronary events such as angina pectoris yet present low risk according to the FRS. Risk prediction is improved by using other markers like CRP in conjunction with the FRS. Koenig et al. found that CRP and FRS used together was better than FRS alone for assessing the risk of coronary events in asymptomatic populations.^4^

Greenstein et al. found a relationship between erectile dysfunction and the presence of coronary disease. There was a positive correlation between the severity of erectile dysfunction and the number of coronary vessels involved.^12^ The presence of coronary calcification, as measured by multislice computed tomography, is directly related to atherosclerotic diseases. Chiurlia showed that the presence and extent of calcification in the coronary arteries is greater in patients with ED.^13^

Giugliano et al. correlated erectile dysfunction with endothelial dysfunction markers through measuring nitric oxide activity. They showed that the nitric oxide response in men with erectile dysfunction was lower than in men without it.^14^

Elesber et al. measured a competitive inhibitor of nitric oxide synthase called asymmetric dimethylarginine (ADMA) to evaluate the presence of endothelial dysfunction among men with erectile dysfunction. In their study, the coronary vasoreactivity was evaluated by measuring dose responses to intracoronary adenosine boluses during coronary angiography. There was a statistically significant correlation between endothelial dysfunction, coronary vasoreactivity, erectile dysfunction and ADMA measurements.^15^

The presence of erectile dysfunction seems to be an early marker of endothelial injury and atherosclerotic disease. Studies have shown that the presence of subclinical coronary disease, as assessed by angiography and multislice tomography, is directly correlated with the severity of erectile dysfunction.^4,16^

Studies have shown that obesity is a predictive factor for erectile dysfunction. Therefore, patients with higher body mass index are at greater risk of erectile dysfunction, even if they lose weight.^11^ Heidler et al. did not find differences in waist-hip ratio greater than 3% in any decade of life between men with and without erectile dysfunction. According to them, the role of the waist-hip ratio in the genesis of erectile dysfunction, in terms of clinical relevance, has to be regarded critically.^10^ In our study, clinical factors like waist-hip ratio and body mass index were significantly higher in patients with erectile dysfunction.

According to Derby et al., men who were overweight were at increased risk of developing ED. They followed up a group of men presenting excess weight and, after two years of a health-related behavior program, regular exercise and weight loss, 33% of the obese men with erectile dysfunction were able to have sexual activities again. Physical activity was associated with a 30% lower risk of erectile dysfunction, while obesity was associated with a 30% higher risk of erectile dysfunction.^17^

Kratzik et al. found a correlation between erectile function and physical activity. The risk of severe erectile dysfunction was 82.9% lower among men with physical activity expending at least 3,000 kcal per week, compared with men with physical activity of under 3,000 kcal per week. The risk of erectile dysfunction may be reduced by increasing physical activity from 1000 to 4000 kcal/week.^18^

Metabolic syndrome affects 10-25% of the adult population worldwide, which is similar to the prevalence that we found in our control group (26.8%). It is an established risk factor for endothelial dysfunction and vascular impairment, and both of these are involved in the pathogenesis of erectile dysfunction. Among the components of metabolic syndrome, fasting blood glucose level, blood pressure, waist circumference and age were the most significant risk factors for predicting the presence of erectile dysfunction.^12^ Kupelian et al. suggested that erectile dysfunction might provide a warning sign and an opportunity for early intervention among men who had previously been considered to present lower risk of metabolic syndrome and cardiovascular disease. Early detection of metabolic disease in patients with erectile dysfunction may be an opportunity for reducing the degree of endothelial dysfunction among younger men presenting higher cardiovascular risk.^9^

A strong association was found between erectile dysfunction and metabolic syndrome. The prevalence of metabolic syndrome in our study among patients with erectile dysfunction was 47.3%. Bansal et al. found similar results: the prevalence of metabolic syndrome in patients with erectile dysfunction was 43% in their study, and it was higher than in patients without erectile dysfunction (24%).^19^ Bal et al. found that the incidence of erectile dysfunction among patients with metabolic syndrome was significantly greater than among those without metabolic syndrome (79% versus 62%). There was a significant age difference between patients with and without metabolic syndrome, but when the patients were stratified according to age group, metabolic syndrome was a significant risk factor for erectile dysfunction only for patients aged 40 to 49 years. In elderly men, the effect of metabolic syndrome on erectile dysfunction was not prominent, probably because older age itself is a strong risk factor for erectile dysfunction.^11^

The importance of recognizing the association between erectile dysfunction and metabolic syndrome is that, by addressing presentations of erectile dysfunction, opportunities for identifying and improving health profiles may be afforded, thereby promoting health maintenance objectives for such patients. Changes to modifiable health-related behavior, such as weight loss and increases in physical activity, may reduce the risk of erectile and endothelial dysfunctions.

## CONCLUSIONS

Men with erectile dysfunction presented higher cardiovascular risk according to Framingham Risk Score and C-reactive protein measurements. Severe erectile dysfunction seemed to have a correlation with metabolic syndrome.
